# Comparative analysis of energy homeostasis regulation at different altitudes in Hengduan Mountain of red-backed vole, *Eothenomys miletus*, during high-fat diet acclimation: examining gut microbial and physiological interactions

**DOI:** 10.3389/fmicb.2024.1434346

**Published:** 2024-07-10

**Authors:** Ting Jia, Wei Zhang, Lijuan Cao, Wanlong Zhu, Lixian Fan

**Affiliations:** ^1^Key Laboratory of Ecological Adaptive Evolution and Conservation on Animals–Plants in Southwest Mountain Ecosystem of Yunnan Province Higher Institutes College, School of Life Sciences, Yunnan Normal University, Kunming, China; ^2^Engineering Research Center of Sustainable Development and Utilization of Biomass Energy Ministry of Education, Yunnan Normal University, Kunming, China; ^3^Key Laboratory of Yunnan Province for Biomass Energy and Environment Biotechnology, Yunnan Normal University, Kunming, China

**Keywords:** *Eothenomys miletus*, high-fat food, body mass regulation, gut microorganisms, different altitudes, adaptive strategy, host–microbe interactions

## Abstract

The study aimed to explore the similarities and differences in gut microorganisms and their functions in regulating body mass in *Eothenomys miletus* across different altitudes in the Hengduan Mountains when exposed to a high-fat diet. *Eothenomys miletus* specimens were gathered from Dali (DL) and Xianggelila (XGLL) in Yunnan Province, China, and categorized into control, high-fat (1 week of high-fat diet), and re-feeding groups (1 week of high-fat diet followed by 2 weeks of standard food). The analysis utilized 16S rRNA sequencing to assess the diversity and structure of intestinal microbial communities in *E. miletus*. The investigation focused on the impact of high-fat diet consumption and different altitudes on gut microbial diversity, structure, and physiological markers. Results revealed that a high-fat diet influenced the beta diversity of gut microorganisms in *E. miletus*, leading to variations in microbial community structure between the two regions with different altitudes. High-fat food significantly affected body mass, white adipose tissue mass, triglycerides, and leptin levels, but not food intake. Specific intestinal microorganisms were observed in the high-fat groups, aiding in food digestion and being enriched in particular flora. In particular, microbial genera like *Lactobacillus* and *Hylemonella* were enriched in the high-fat group of DL. The enriched microbiota in the control group was associated with plant polysaccharide and cellulose decomposition. Following a high-fat diet, gut microbiota adapted to support lipid metabolism and energy supply, while upon re-feeding, the focus shifted back to cellulose digestion. These findings suggested that alterations in gut microbial composition, alongside physiological markers, play a vital role in adaptation of *E. miletus* to the diverse habitats of the Hengduan Mountains at varying altitudes.

## 1 Introduction

During long-term co-evolution, the host provides the living environment and nutrients for the gut microbes, which can assist the host in digesting food, producing short-chain fatty acids (SCFAs) and other metabolites, participating in physiological processes, such as individual development, nutrient absorption, energy metabolism, or immune responses, which play an important role in the adaptation of host under environmental changing (Sender et al., 2016; Moran et al., 2019). The makeup and variety of microorganisms in the gastrointestinal tract are impacted by factors such as the dietary choices of the host, their genetic predisposition, and the environmental conditions of their habitat (Carmody et al., 2015; Thaiss et al., 2016). The nutritional makeup of a diet is acknowledged as a significant factor influencing the diversity of gut microbiota, leading to substantial variations in the microbial populations of mammals following different dietary patterns (Amato et al., 2013; Guo et al., 2021). As the environmental food changes, the gut microorganisms adjust accordingly as follows: *Apodemus sylvaticus* shifts its food from insectivorous to seed-feeding during seasonal changes, its gut microbial community showed a gradual decrease in the content of *Lactobacillus*, and a significant increase in the content of *Helicobacter* and *Alistipes* during the food transition (Maurice et al., 2015). In their native habitats, animals consume a diverse array of food sources in order to fulfill their daily energy and nutritional needs (Bolnick et al., 2014). Studying the relationship between gut microbiota and diet in animals can help our understanding of the multidirectional interaction between microorganisms, hosts, and the environment, providing more evidence to reveal the different adaptations of animals to the environment.

The lipid composition of dietary items plays a significant role in the accumulation of adipose tissue, and the consumption of high-fat products in conjunction with surplus caloric intake may result in atypical physical structure and have adverse effects on general wellbeing and immune function (Neyrinck et al., 2016). Various nutrients can offer distinct nutritional ecological niches that are capable of sustaining diverse microbial communities (Tan and Norhaizan, 2019). The gut microbiota is closely related to the digestion, absorption, and energy metabolism of food ingredients, and hence, intestinal microorganisms will affect energy absorption and fat storage (Tremaroli et al., 2010). Studies have shown that the consumption of high-fat foods leads to rapid and continuous changes in the gut microbiota of both mice and humans within a timeframe of 24 to 48 h (David et al., 2014). High-fat foods promoted lipid accumulation, and gut microbiota diseases in mice reduced the diversity of the gut microbiota, reduced its SCFAs content and the number of beneficial bacteria, and increased the number of pathogenic bacteria (Yin et al., 2018; Kong et al., 2019). Furthermore, the consumption of high-fat foods led to an elevation in the presence of lipopolysaccharide (LPS) generating bacteria within the intestinal tract, resulting in the upregulation of tumor necrosis factor α (TNF-α) expression in the ileum of mice (Konrad and Wueest, 2014). LPS and TNF-α inhibited adipose tissue browning, causing a decrease in energy expenditure and indirectly leading to obesity and related metabolic diseases (Lucchini et al., 2020). Consumption of high-fat foods led to a notable decrease in the prevalence of Bacteroidetes and a significant increase in the prevalence of Firmicutes in the gastrointestinal tract of animals (Zhang and Yang, 2016). Some studies have also shown that high-fat food increased the relative abundance of Bacteroidota; for example, high-fat food significantly increased the relative abundance of Bacteroidota in male C57BL/6 mice and significantly reduced the relative abundance of Actinobacteria and Firmicutes (Zhou et al., 2017). At present, the existing research studies pertaining to the impact of high-fat diets on host physiology and gut microbial diversity remain inconclusive.

Hengduan mountain regions are regions of higher climatic and geographic diversity, and the red-backed vole (*Eothenomys miletus*) is an inherent species of this region (Gong et al., 2001; Zhu et al., 2014a,b). At present, it has been confirmed that temperature, photoperiod, and food quantity or quality are major ecological variables that affect the energy metabolism of *E. miletus* (Zhu et al., 2010; Zhu W. L. et al., 2011). Seasonal differences in thermogenesis and metabolism of *E. miletus* may be related to differences in food resources in different seasons, and significant differences in body mass were found when treated with different food conditions (Zhu et al., 2014a). It has been proposed that the consumption of high-fat food has a notable impact on body mass, food consumption, and resting metabolic rate (RMR) in *E. miletus*, leading to significant variations in the expression of leptin (Gong et al., 2021). Our research discovered variations in the intestinal microorganisms of *E. miletus* across different regions of the Hengduan mountains, such as Dali, Jianchuan, Lijiang, Deqin, and Xianggelila (Yan et al., 2022). Dali (DL) and Xianggelila (XGLL), the southernmost and northernmost points of the Hengduan Mountains in Yunnan Province, exhibit distinctive environmental conditions, making them ideal study sites to effectively showcase the adaptation of *E. miletus* to the Hengduan Mountain region. Based on previous research, the present study was conducted to study the variation in the influence that high-fat food has on the gut microorganisms and body mass adjustment of *E. miletus* between two regions with different altitudes, DL (low altitude region) and XGLL (high altitude region) in winter, and to ultimately elucidate the relationship between the gut microorganisms and the body mass regulation.

## 2 Materials and methods

### 2.1 Collection of experimental animals

*Eothenomys miletus* from DL and XGLL were collected in the winter of 2022, respectively. The experimental animals were all non-breeding healthy adult individuals. The positions, climates, and sample data of the sampling sites are shown in Table 1.

**Table 1:** Detailed information on the sampling sites in *Eothenomys miletus.*

Regions	Sample size	Geographic location	Altitude/m	Average temperatures (winter)/°C	Precipitation/mm
XGLL	20(♂10, ♀10)	99°83′16″ E, 27°90′73″ N	3,321	4.5	984.2
DL	19(♂10, ♀9)	100°42′49″ E, 24°90′30″ N	2,217	17.5	597.0

XGLL, Xianggelila; DL, Dali.

### 2.2 Experimental designs

*Eothenomys miletus* were captured from two sites and brought to Yunnan Normal University for rearing in the animal breeding room through sterilization and flea killing. After 4 days of acclimatization in the laboratory, specimens of *E. miletus* from DL and XGLL with different altitudes were chosen for the experiment. The experiment involved high-fat foods and different regions as variables, with *E. miletus* specimens from each region being categorized into a control group (Con, 0 day of execution), high-fat group (HF, provided with high-fat food for 1 week), and high-fat re-feeding group (Re, given high-fat food for 1 week followed by a return to standard food for 2 weeks). These groups were designated as DLC, XGC, DLHF, XGHF, DLRe, and XGRe, respectively. The experiment was conducted for a period of 3 weeks at a room temperature of 25 ± 1°C and an environmental photoperiod condition of 12 L:12D (light: dark). During the experimental period, *E. miletus* had free access to food and water and were fed standard diets and high-fat diets produced by Kunming Medical University (Table 2). Body mass, food intake, and RMR were measured on days 0, 7, and 21, respectively, measurement methods as described by Steelman et al. (2012) and Zhu et al. (2012). Finally, *E. miletus* was euthanized, which was reported in accordance with ARRIVE guidelines (Zhu et al., 2008); then, serum was taken, and rectal feces were obtained.

**Table 2:** Food ingredients.

Contents	Standard diet	High-fat diet
Crude fat (%)	6.2	21.4
Crude protein (%)	20.8	17.6
Neutral detergent fiber (%)	21.5	19.6
Acid detergent fiber (%)	12.5	10.6
Ash (%)	10.0	8.5
Caloric value (kJ/g)	17.5	19.7

### 2.3 Measurement of physiological indices

At the end of the experiment for each group, blood was collected, allowed to stand for 1 h in the refrigerator at 4°C, and was centrifuged at 4°C (4,000 r/min, 30 min), taking up serum in a centrifuge tube, stored in the refrigerator (−80°C), and set aside. Measurements of leptin, glucose (Glu), triglyceride (Tg), total cholesterol (Tc), SCFAs, LPS, fasting-induced adipocyte factor (FIAF), and TNF-α were measured by the serum using an enzyme-linked immunosorbent assay (ELISA). After the experimental animals were executed, brown adipose tissue (BAT) was carefully removed, weighed and put into centrifuge tubes, and stored in a refrigerator (−80°C) for storage. The content of uncoupling protein 1 (UCP1) was determined using ELISA. The assay was conducted following the instruction manual, and the product numbers of the assay kits are listed in Table 3.

**Table 3:** Kit numbers.

Kit name	Product number
Leptin ELISA kit	JM-11498 M1
Glu ELISA kit	S0104F-1
Tg ELISA kit	S0140O-1
Tc ELISA kit	S05042-1
SCFAs ELISA kit	JM-11498 M1
LPS ELISA kit	M-12488 M1
FIAF ELISA kit	JM-12613 M1
TNF-α ELISA kit	JM-02415 M1
UCP1 ELISA kit	JM-12185 M1

### 2.4 Measurement of digestive tract morphology

After separating the organs and removing other tissues, weigh after draining the surface liquid using filter paper (accurate to 0.001 g). Remove the digestive tract, to separate the stomach, small intestine, large intestine, and cecum, remove carefully the mesentery and connective tissue and fat of each organ, and then weigh and measure the length.

### 2.5 DNA extraction and 16S rRNA gene sequencing

Rectal feces were collected, and the total DNA was enriched on the filter membrane using a centrifugal column-based soil genome extraction kit (DNeasy®PowerSoil®Kit, Germany). The purified DNA samples were subjected to sequencing on the Illumina MiSeq platform (Illumina, San Diego, CA, United States), which was operated by Beijing Novozymes Co.

### 2.6 Bioinformatics analysis

By QIIME software, the raw data were processed, using Flash software to clear low-quality sequences, and then removing the chimeras in the sequences by Usearch 7.0 software. The OTU sequences with more than 97% recognition were clustered using the Uclust algorithm, and the representative OTU sequences were analyzed and identified based on the Ribosomal Database Project, finally, the sequences of all samples were normalized by the “Daisychopper” script code (Zhu L. et al., 2011; Percie du Sert et al., 2020). Finally, we standardized the sequences of all the samples by using the code “Daisychopper,” and the standard of each sample was 5,437 sequences.

### 2.7 Data analysis

#### 2.7.1 Microbiology-related analyses

Two diversity indices, Chao1 and Shannon diversity, were employed for the evaluation of α diversity; β diversity was assessed through the utilization of unweighted and weighted UniFrac distance matrices. Graphical representations of the pertinent metrics have been generated using Origin 2018. Venn diagrams were drawn using Venn 2.1 to describe common and gap flora between groups.^1^ To analyze the enriched flora of each group, one-way analysis of variance (ANOVA) method was used. Heat maps of dominant bacteria and correlation of physiological and serum biochemical indices were obtained using Pearson analysis with SPSS 21 and R3.6.2. Canoco 5.0 was used to assess the correlation between dominant genera and physicochemical factors using redundancy analysis (RDA). These results were further analyzed using R3.6.2 and Gephiv.0.9.2 software to generate network analyses (*p* < 0.05, |r| > 0.4).

#### 2.7.2 Physiological indicator analysis

To analyze the data, SPSS 26.0 software (SPSS Inc., Chicago, IL, United States) was used. Differences in measurement indicators between the different sexes in *E. miletus* were not significant, so all data were combined and counted. Moreover, two-way ANOVA or two-way ANCOVA was used to analyze the variability of the different indicators between the two regions with body mass as a covariate. It is expressed as mean ± standard error (mean ± SE), where *p* < 0.05 is considered as significant difference.

## 3 Results

In the present study, a total of 39 samples were collected to extract the DNA and amplify the PCR products, which were normalized to 8,296 sequences per sample after eliminating low-quality sequences, chimeras, monomers, and chloroplasts.

### 3.1 Microbial community composition

**Figure 1:**
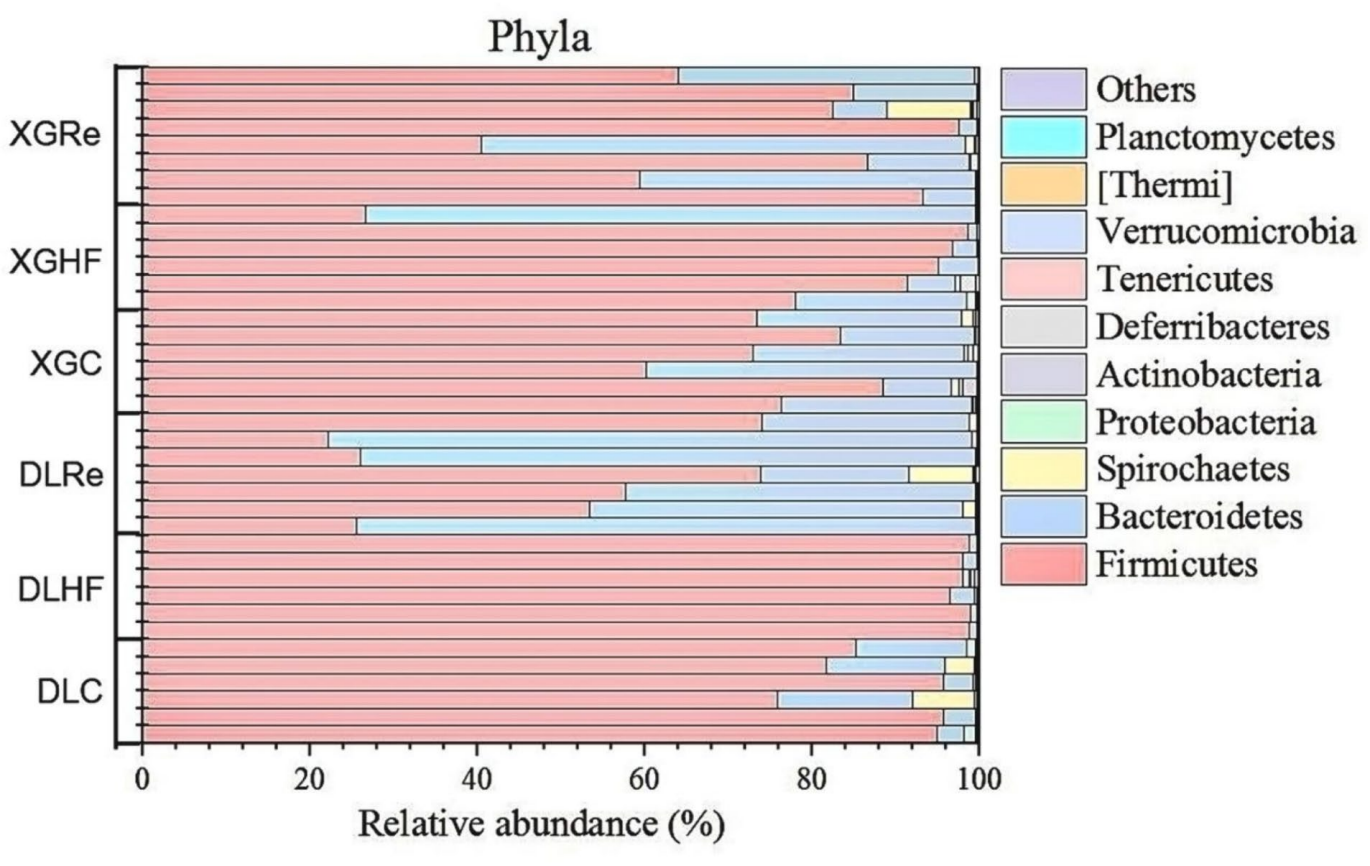
Community composition at the microbial phylum level. DLC, Dali control group; DLHF, Dali high-fat group; DLRe, Dali high-fat re-feeding group; XGLC, Xianggelila control group; DXGHF, Xianggelila high-fat group; XGRe, Xianggelila high-fat re-feeding group.

**Figure 2:**
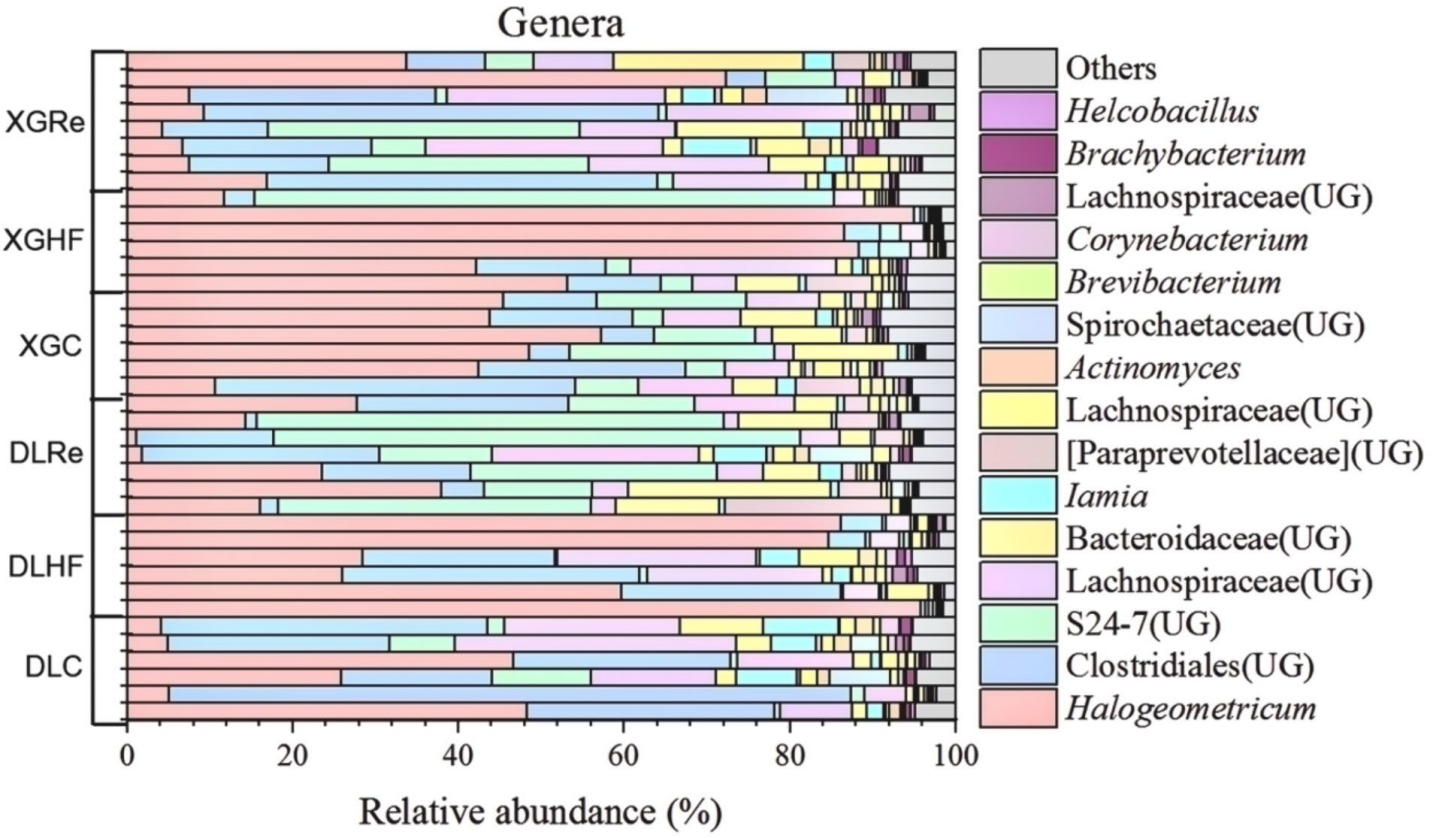
Community composition at the genus level of microorganisms. DLC, Dali control group; DLHF, Dali high-fat group; DLRe, Dali high-fat re-feeding group; XGLC, Xianggelila control group; DXGHF, Xianggelila high-fat group; XGRe, Xianggelila high-fat re-feeding group.

On the level of phylum, Firmicutes, Bacteroidetes, and Spirochaetes were the dominant fecal microbial phylum in two different elevation areas of *E. miletus*, with mean relative abundances within all groups of 76.94%, 21.39%, and 0.92%, respectively. It had a higher proportion of Bacteroidetes in the DLRe group than in the other two groups, and a higher proportion of Firmicutes/Bacteroidetes in the DLHF group compared to the other two groups. Spirochaetes had higher proportions in the control and Re groups than in the HF group, and the proportion of Firmicutes was relatively higher in the HF group than in the other two groups in both two regions (Figure 1). The major genera of *Hologeometricum*, *Clostridiales (UG)*, and S24-7(UG) were the dominant genera of fecal microorganisms of *E. miletus* at the genus level, with mean relative abundances within all groups of 36.39%, 19.52%, and 12.96%, respectively. *Halogeometricum* was relatively higher in the HF group than in the control and Re groups, and S24-7(UG) was lower in the HF group relative to the other two groups (Figure 2).

### 3.2 Microbial community alpha diversity analysis

**Figure 3:**
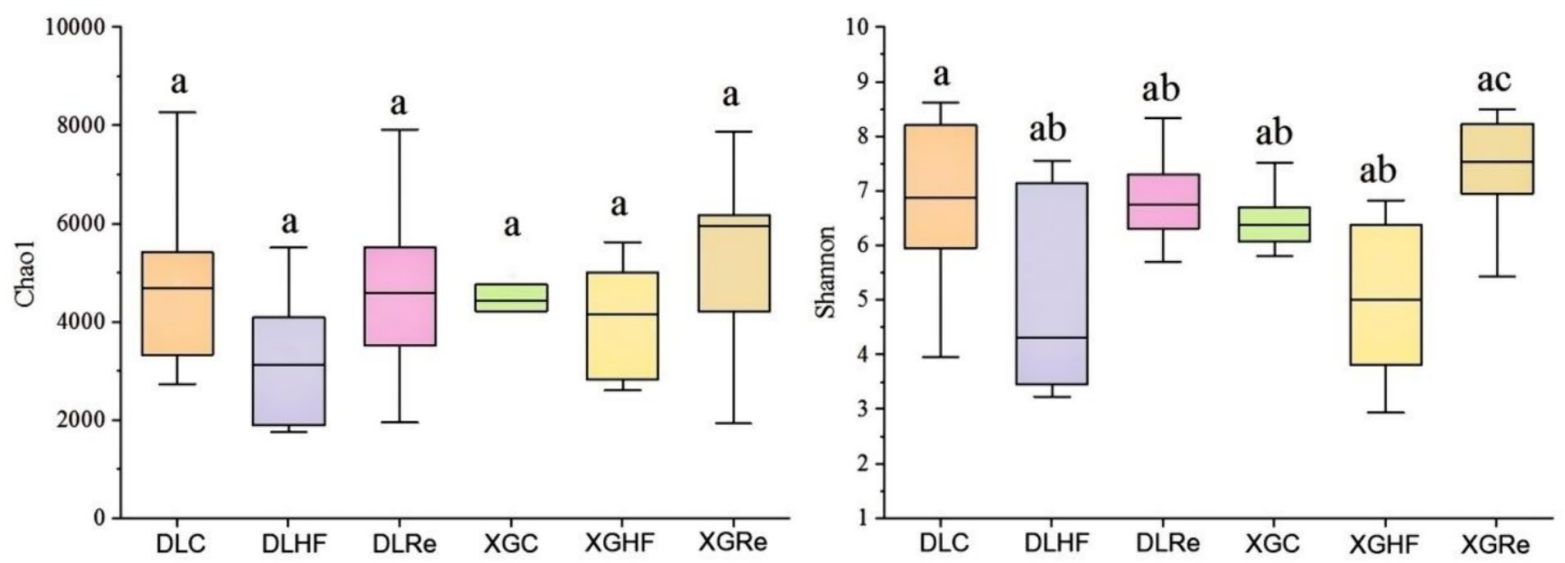
Microbial α-diversity in different groups. DLC, Dali control group; DLHF, Dali high-fat group; DLRe, Dali high-fat re-feeding group; XGLC, Xianggelila control group; DXGHF, Xianggelila high-fat group; XGRe, Xianggelila high-fat re-feeding group. Different letters indicate significant differences between groups.

Chao1 diversity was found to be not significantly different between two different altitudes (*p* > 0.05). The Shannon diversity of fecal microorganisms in *E. miletus* of the XGRe group was significantly different from the DLHF and XGHF groups, which was lower than that of the DLHF and XGHF groups (*p* < 0.05, Figure 3).

### 3.3 Microbial community β diversity analysis

**Figure 4:**
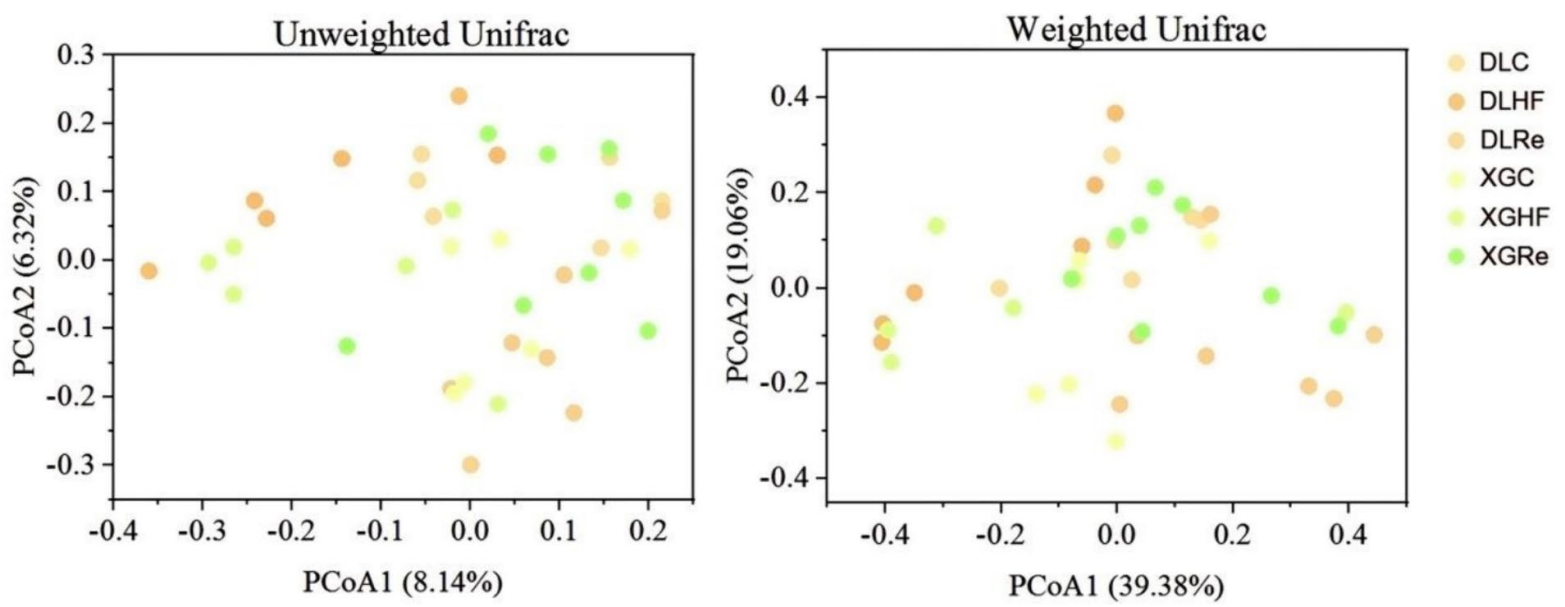
Microbial β-diversity in different groups. DLC, Dali control group; DLHF, Dali high-fat group; DLRe, Dali high-fat re-feeding group; XGLC, Xianggelila control group; DXGHF, Xianggelila high-fat group; XGRe, Xianggelila high-fat re-feeding group.

There was no significant trend of aggregation of fecal microbial β diversity on PCoA plots in two regions of the HF group in *E. miletus* (Figure 4). However, the overall distribution of fecal microbial β diversity (weighted and unweighted matrices) was found to be significantly different (*p* < 0.05) in different regions of the HF group according to the PERMANOVA test (Table 4). Additional PERMANOVA indicated that there was no statistically significant variation (*p* > 0.05) in the fecal microbial β diversity among regions within the same timeframe. However, there were notable alterations (*p* < 0.05) in β diversity over time within the same region. Specifically, there were significant shifts in microbial community composition observed from the control group to the high-fat (HF) group and from the HF group to the Re group.

**Table 4:** Microbiological PERMANOVA test for feces of *Eothenomys miletus* feces.

PERMANOVA	Unweighted Unifrac			Weighted Unifrac		
	*F*	*R*2	*P*	*F*	*R*2	*P*
DLC vs. DLHF	2.753	0.216	0.029	3.784	0.275	0.056
DLHF vs. DLRe	4.645	0.297	0.002	4.539	0.292	0.002
XGC vs. XGHF	2.886	0.224	0.014	3.090	0.236	0.038
XGHF vs. XGRe	3.732	0.237	0.005	5.403	0.310	0.004
DLC vs. XGC	1.745	0.149	0.075	2.405	0.194	0.077
DLHF vs. XGHF	0.938	0.086	0.438	0.351	0.034	0.865
DLRe vs. XGRe	1.377	0.096	0.163	2.440	0.158	0.103

*P* < 0.05 indicates a significant difference.DLC, Dali control group; DLHF, Dali high-fat group; DLRe, Dali high-fat re-feeding group; XGLC, Xianggelila control group; DXGHF, Xianggelila high-fat group; XGRe, Xianggelila high-fat re-feeding group.

### 3.4 Distribution of common and unique microorganisms in different altitudes and periods

**Figure 5:**
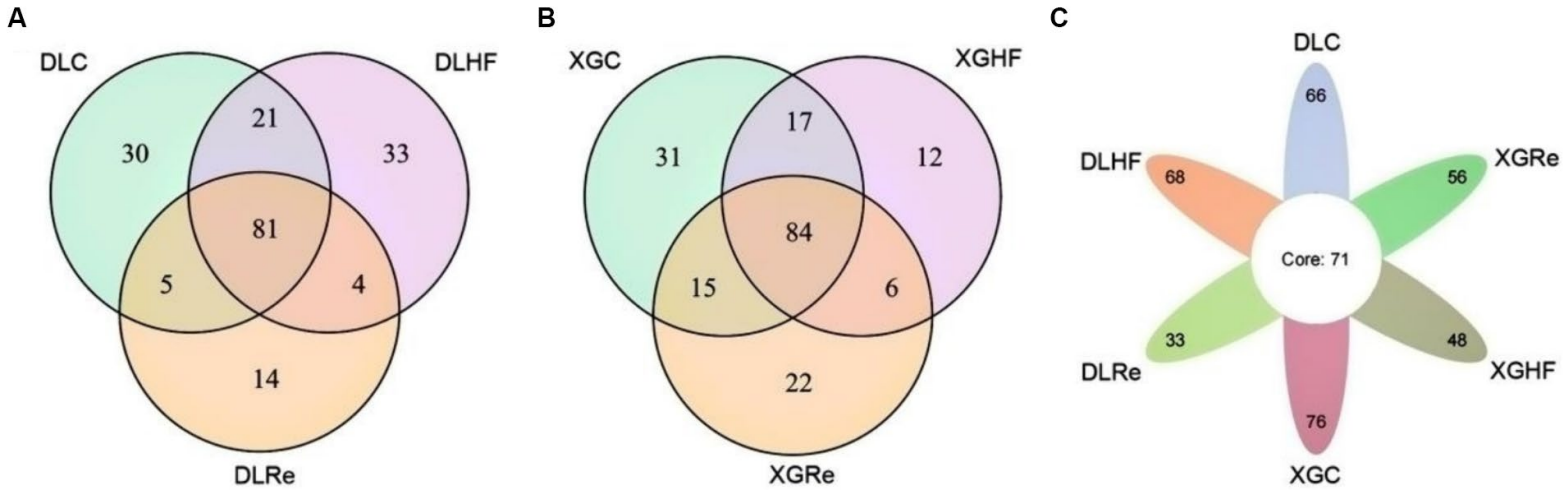
Venn diagrams of microorganisms in different groups. DLC, Dali control group; DLHF, Dali high-fat group; DLRe, Dali high-fat re-feeding group; XGLC, Xianggelila control group; DXGHF, Xianggelila high-fat group; XGRe, Xianggelila high-fat re-feeding group.

In DL, the total number of microorganisms in the feces of *E. miletus* was 81 genera. The fecal microorganisms of the DLC and the DLHF groups contained more unique genera than those of the DLRe group. The number of common genera of fecal microorganisms in the XGLL region was 84, which was lower in the XGHF group than in the other two groups. Among the fecal microorganisms from different regions and food conditions, 71 genera were found in the feces of *E. miletus*. DLHF group had a higher number of unique genera than the XGHF group, but the DLC and the DLHF groups had a lower number of unique genera than the XGC group and the XGHF group, with the XGC group had the highest number of unique genera (Figure 5).

### 3.5 Analysis of microbial enrichment differences in different altitudes and periods

**Figure 6:**
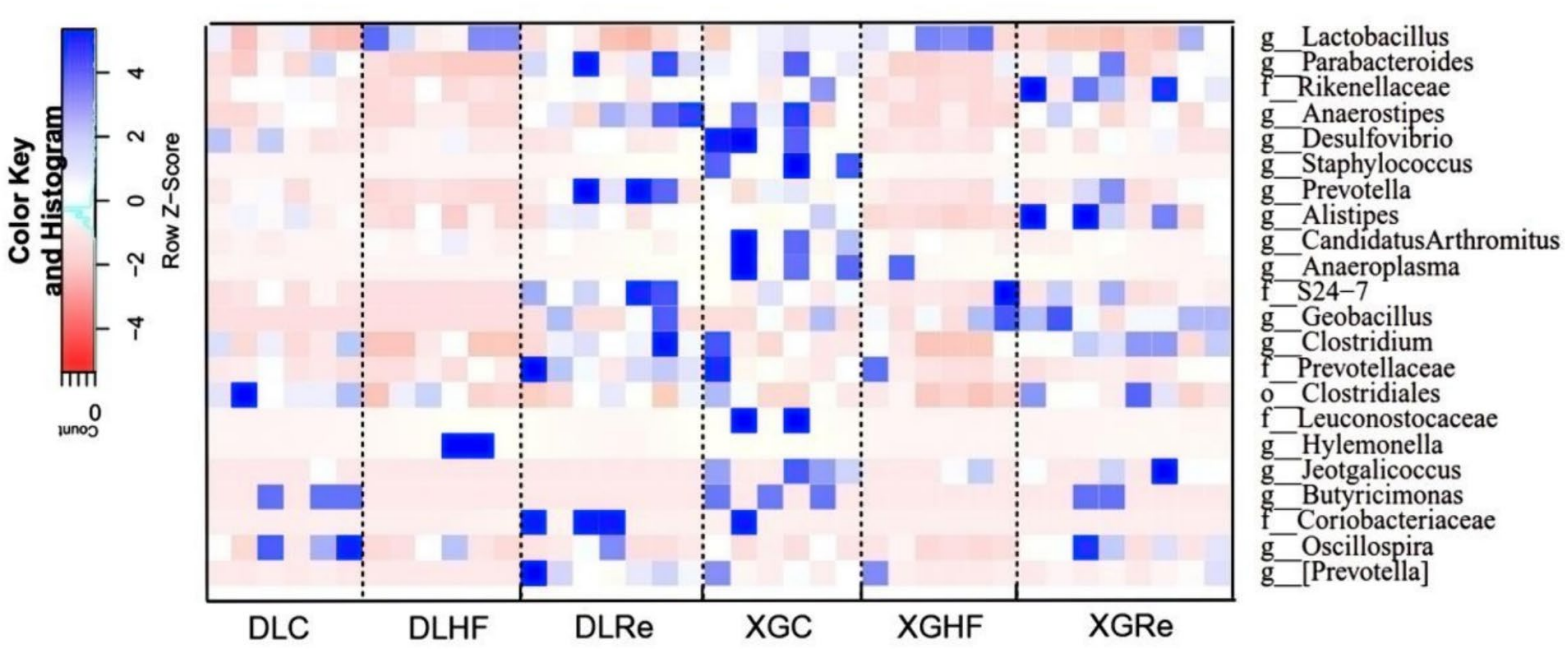
Analysis of microbial differences in different groups. DLC, Dali control group; DLHF, Dali high-fat group; DLRe, Dali high-fat re-feeding group; XGLC, Xianggelila control group; DXGHF, Xianggelila high-fat group; XGRe, Xianggelila high-fat re-feeding group.

The relative abundance of microorganisms differed between DL and XGLL as shown in Figure 6. Microbial genera enriched in the DLC group included *Clostridium*, *Oscillospira,* and *Butyricimonas* (*p* < 0.05). Microbial genera enriched in the DLHF group included *Lactobacillus* and *Hylemonella* (*p* < 0.05). Genera such as *Parabacteroides* and *Prevotella* were significantly enriched in the feces of the DLRe group compared to the DLC and DLHF groups (*p* < 0.05). The XGC group showed a significantly different enrichment distribution from the XGHF and the XGRe groups, with the enrichment of *Anaerostipes*, *Desulfovibrio*, *Candidatus Arthromitus*, *Anaeroplasma,* and *Butyricimomas*. *Anaerostipes* and *Desulfovibrio* enriched significantly (*p* < 0.05) in the XGRe group as compared to the XGHF and XGC groups (Figure 6).

### 3.6 Effects of high-fat foods and altitudes on physiological indicators

It found that high-fat food had a significant effect on body mass, food calories, and WAT mass of *E. miletus* (body mass: *F* = 10.493, *p* < 0.001; food calorie: *F* = 3.487, *p* < 0.05; WAT mass: *F* = 12.269, *p* < 0.001), and the body mass and WAT mass in the HF group were significantly higher than that in the other two groups (Table 5). Tg and leptin were significantly higher in the HF group than in the other groups (Tg: *F* = 6.960, *p* = 0.003; leptin: *F* = 28.657, *p* < 0.001; Table 6). Further analyses revealed that heart mass in the XGLL region differed significantly between the HF and Re groups, with lower heart mass in the HF group (Table 5). In XGLL, TNF-α was significantly affected by high-fat food, which was significantly higher in the HF group than in the other groups; meanwhile, Tc was significantly affected by high-fat food in DL (Table 6).

**Table 5:** Physiologic indices in *Eothenomys miletus* as affected by high-fat food and different altitude.

Parameter	DL			XGLL		
	Con group	HF group	Re group	Con group	HF group	Re group
	*n* = 6	*n* = 6	*n* = 7	*n* = 6	*n* = 6	*n* = 8
Body mass (g)^*1^	36.59 ± 2.06	39.10 ± 1.87	35.78 ± 1.80	32.16 ± 2.17	34.90 ± 2.64	31.43 ± 1.25
Food intake (g/d)^*^	6.31 ± 1.02	5.77 ± 0.79	6.85 ± 0.35	8.17 ± 1.06	8.69 ± 0.74	8.80 ± 0.55
Food calories (kJ/d)^*1^	110.37 ± 17.87	113.63 ± 15.62	119.93 ± 6.14	142.95 ± 18.51	171.10 ± 14.65	155.83 ± 17.55
RMR (mLO2/g.h)^*^	1.91 ± 0.23	1.69 ± 0.49	1.93 ± 0.23	2.72 ± 0.36	2.92 ± 0.16	2.87 ± 0.43
Heart mass (g)	0.24 ± 0.04	0.27 ± 0.06	0.28 ± 0.03	0.29 ± 0.02	0.27 ± 0.04	0.33 ± 0.03
Liver mass (g)^*^	1.85 ± 0.15	1.84 ± 0.27	1.92 ± 0.15	2.48 ± 0.31	2.20 ± 0.17	2.42 ± 0.17
Spleen mass (g)	0.07 ± 0.016	0.07 ± 0.02	0.07 ± 0.02	0.11 ± 0.04	0.08 ± 0.03	0.10 ± 0.04
Kidney mass (g)	0.54 ± 0.13	0.55 ± 0.18	0.55 ± 0.12	0.54 ± 0.13	0.66 ± 0.21	0.60 ± 0.18
Lung weight (g)	0.30 ± 0.06	0.28 ± 0.02	0.27 ± 0.02	0.27 ± 0.04	0.31 ± 0.06	0.30 ± 0.04
Small intestine length (cm)	37.72 ± 4.55	39.39 ± 2.77	39.62 ± 4.24	40.49 ± 6.10	43.06 ± 7.31	41.00 ± 8.61
Cecal length (cm)^*^	8.92 ± 0.31	8.70 ± 0.24	9.24 ± 0.46	10.11 ± 0.89	10.15 ± 0.60	10.36 ± 0.35
WAT mass (g)^*1a^	0.45 ± 0.07	0.49 ± 0.10	0.45 ± 0.08	0.30 ± 0.10	0.49 ± 0.06	0.45 ± 0.05
BAT mass (g)^*^	0.17 ± 0.06	0.18 ± 0.04	0.17 ± 0.04	0.23 ± 0.03	0.28 ± 0.06	0.30 ± 0.05

Data are presented as mean ± SE; ^*^Means that there is a significant difference in the impact of the indicator by altitude.^1^Means that there is a significant difference in the impact of the indicator by high-fat food.^a^Means that there is a significant difference in the interaction between altitude and high-fat food on the indicator; XGLL, Xianggelila; DL, Dali.

**Table 6:** Serum physiological indices in *Eothenomys miletus* as affected by high-fat food and different altitudes.

Parameter	DL			XGLL		
	Con group	HF group	Re group	Con group	HF group	Re group
	*n* = 6	*n* = 6	*n* = 7	*n* = 6	*n* = 6	*n* = 8
Tg (μg/g)^*1^	4001.35 ± 278.66	4520.28 ± 206.11	4093.16 ± 215.59	3399.26 ± 225.32	3733.27 ± 252.03	3451.32 ± 255.57
Tc (μg/g)	1875.87 ± 144.65	2196.83 ± 154.29	1826.61 ± 272.91	1739.56 ± 117.02	1853.08 ± 159.69	1715.59 ± 263.38
Glu (mg/mL)	0.65 ± 0.20	0.73 ± 0.08	0.65 ± 0.11	0.62 ± 0.13	0.66 ± 0.09	0.62 ± 0.10
Leptin (pg/mL)^*1a^	1529.46 ± 126.04	1959.12 ± 109.03	1589.15 ± 100.73	1033.89 ± 130.95	1222.42 ± 99.67	1023.46 ± 70.88
UCP1(pg/mL)^*^	1222.23 ± 74.58	1201.65 ± 80.52	1293.84 ± 119.04	1496.30 ± 175.43	1407.05 ± 65.50	1495.23 ± 117.71
SCFAs (μmol/L)^*^	32.46 ± 7.31	29.50 ± 5.59	34.11 ± 5.14	33.85 ± 7.23	33.08 ± 7.57	33.09 ± 3.02
FAIF (ng/L)	55.26 ± 7.67	64.32 ± 12.70	55.01 ± 8.99	61.93 ± 10.53	66.84 ± 10.42	60.61 ± 13.04
LPS (ng/mL)^*^	11.59 ± 1.96	12.05 ± 2.03	12.03 ± 1.109	11.75 ± 1.32	11.21 ± 1.11	11.74 ± 1.31
TNT-α (ng/L)	1075.21 ± 129.91	1124.03 ± 113.54	1070.69 ± 148.44	1078.89 ± 86.04	1205.62 ± 90.94	1028.90 ± 78.07

Data are presented as mean ± SE; *Means that there is a significant difference in the impact of the indicator by altitude.^1^Means that there is a significant difference in the impact of the indicator by high-fat food.^a^Means that there is a significant difference in the interaction between of altitude and high-fat food on the indicator; XGLL, Xianggelila; DL, Dali; Glu, glucose; Tg, triglyceride; Tc, total cholesterol; SCFAs, short-chain fatty acids; LPS, lipopolysaccharide; FIAF, fasting-induced adipocyte factor; TNF-α, tumor necrosis factor-α.

It was found that the body mass, food intake, food calories, RMR, liver mass, cecum length, WAT mass, and BAT mass of *E. miletus* were significantly different between the two regions (body mass: *F* = 45.590, *p* < 0.001; food intake: *F* = 31.353, *p* < 0.001; food calories: *F* = 32.123, *p* < 0.001; RMR: *F* = 25.574, *p* < 0.001; liver mass: *F* = 20.373, *p* < 0.001; cecum length: *F* = 22.114, *p* < 0.001; WAT mass: *F* = 12.404, *p* = 0.001; BAT weight: *F* = 18.926, *p* < 0.001), and other indexes except body mass and WAT mass were significantly higher in DL than in XGLL (Table 5). Tg and leptin were significantly higher in DL than in XGLL (Tg: *F* = 30.008, *p* < 0.001, leptin: *F* = 164.165, *p* < 0.001; Table 6). The SCFAs and UCP1 were significantly affected by different altitudes (SCFAs: *F* = 5.582, *p* = 0.024, UCP1: *F* = 19.276, *p* < 0.001), and both were significantly more abundant in XGLL than in DL (Table 6). Further analyses revealed significant differences in Tc in the HF groups in both regions (Table 6). Moreover, the interaction of high-fat food and region was significantly affected by WAT mass and leptin in *E. miletus* (WAT mass: *F* = 5.215, *p* = 0.011; leptin: *F* = 4.221, *p* = 0.024).

### 3.7 Relationship between physiological indicators and microorganisms in different altitudes and periods

**Figure 7:**
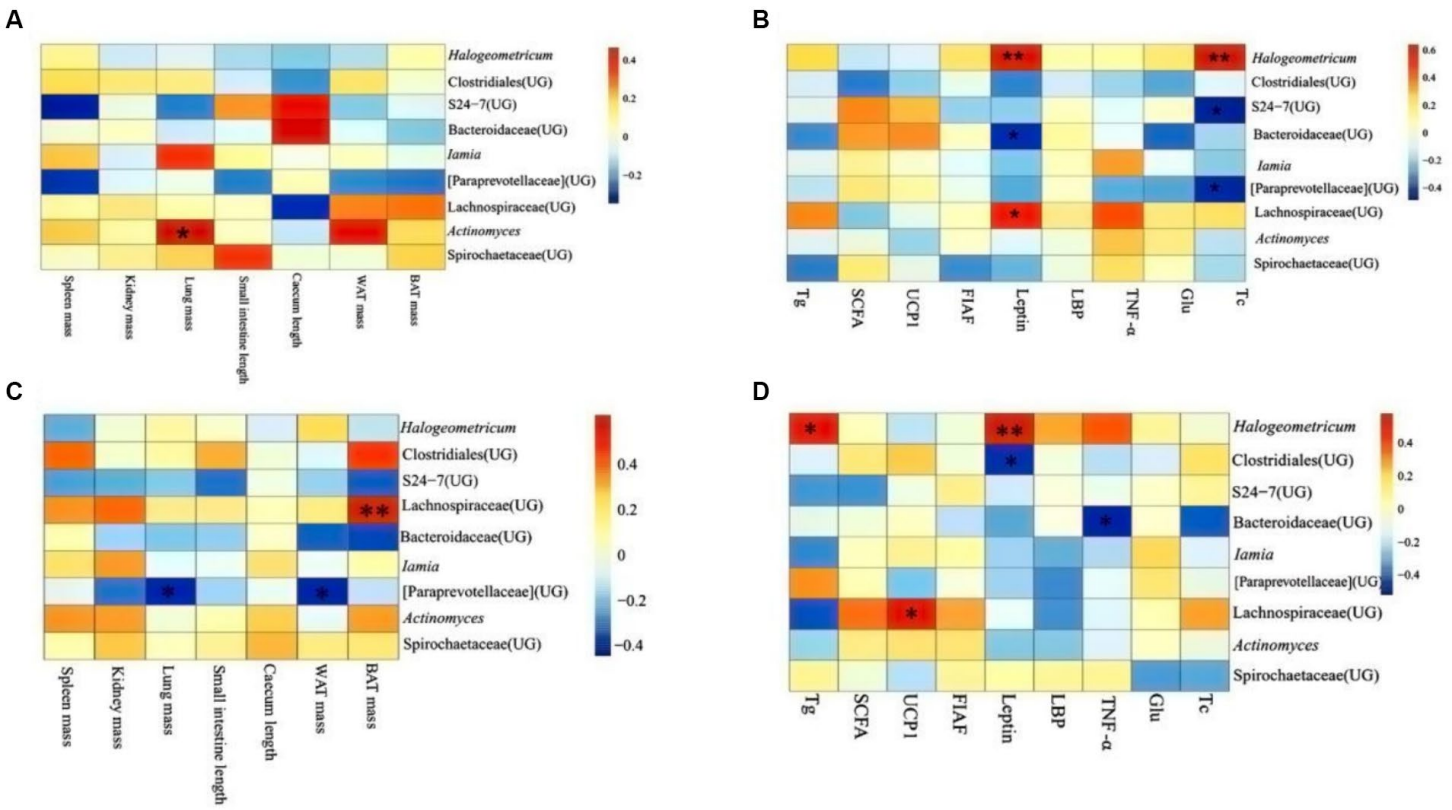
Heat map of physiological indicators associated with dominant microorganisms in different regions. **(A)** Indicators of physiology in Dali. **(B)** Indicators of serum physiology in Dali. **(C)** Indicators of physiology in Xianggelila. **(D)** Indicators of serum physiology in Xianggelila. Glu, glucose; Tg, triglyceride; Tc, total cholesterol; SCFAs, short-chain fatty acids; LPS, lipopolysaccharide, FIAF, fasting-induced adipocyte factor; TNF-α, tumor necrosis factor-α. ^*^Means significant impact, ^**^Means highly significant impact.

In DL, it revealed that lung mass correlated significantly and positively with the abundance of *Actinomyces* (*p* < 0.05; Figure 7A). Leptin was positively related to the abundance of *Hologeometricum* and *Lachnospiraceae* (UG) and negatively related to the abundance of *Bacteroidaceae* (UG; *p* < 0.05). Moreover, Tc positively related to the abundance of *Hologeometricum* and negatively related to S27-4 (UG) and *Paraprevotellaceae* (UG; *p* < 0.05; Figure 7B).

In XGLL, it was found the BAT mass and *Lachnospiraceae* (UG) abundance correlated positively (*p* < 0.05); lung weight and WAT mass related dramatically and negatively to *Paraprevotellaceae* (UG) abundance (*p* < 0.05; Figure 7C). *Hologeometricum* abundance was significantly positively correlated with Tg and Leptin (*p* < 0.05); a significant positive correlation was found between *Lachnospiraceae* (UG) and UCP1 (*p* < 0.05); and a significant negative correlation was found between *Bacteroidaceae* (UG) and TNF-α (*p* < 0.05, Figure 7D).

**Figure 8:**
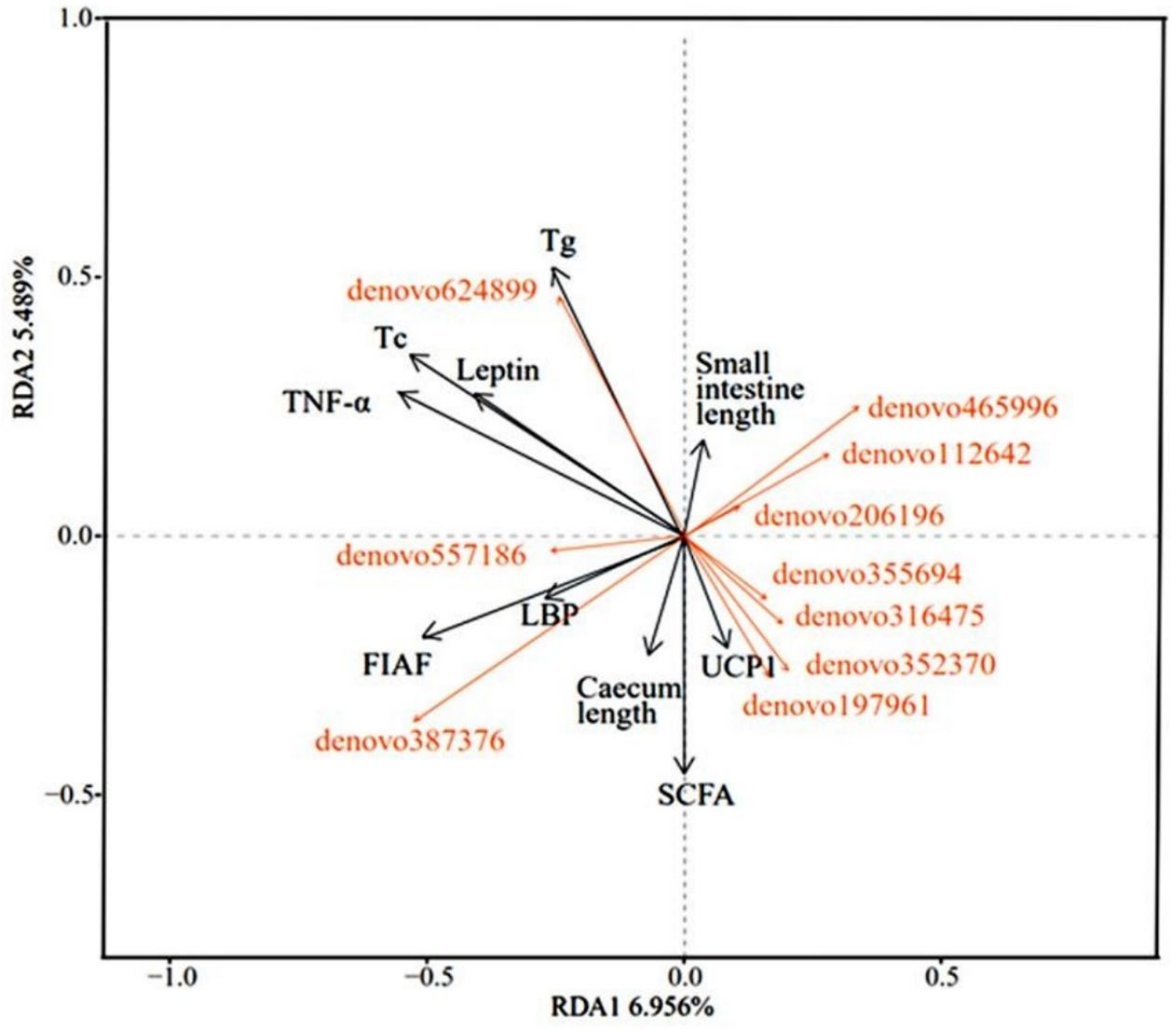
Redundancy analysis of correlations between physiological indicators and dominant microbial communities.

The analysis of the relevance of physiological indicators to microbial dominance OTUs in DL and XGLL of *E. miletus* showed positive associations among Tg, Tc, leptin, TNF-α, and denovo624899 abundance. Positive correlations were found between small intestine length and denovo465996, denovo112642, and denovo206196. There were positive correlations between UCP1 and denovo197961, denovo352370, denovo316475, and denovo355694. The length of the cecum, LBP, and FIAF was positively correlated with denovo 387,376 and denovo 557,186 (Figure 8).

### 3.8 The fecal microbial co-occurrence network

**Figure 9:**
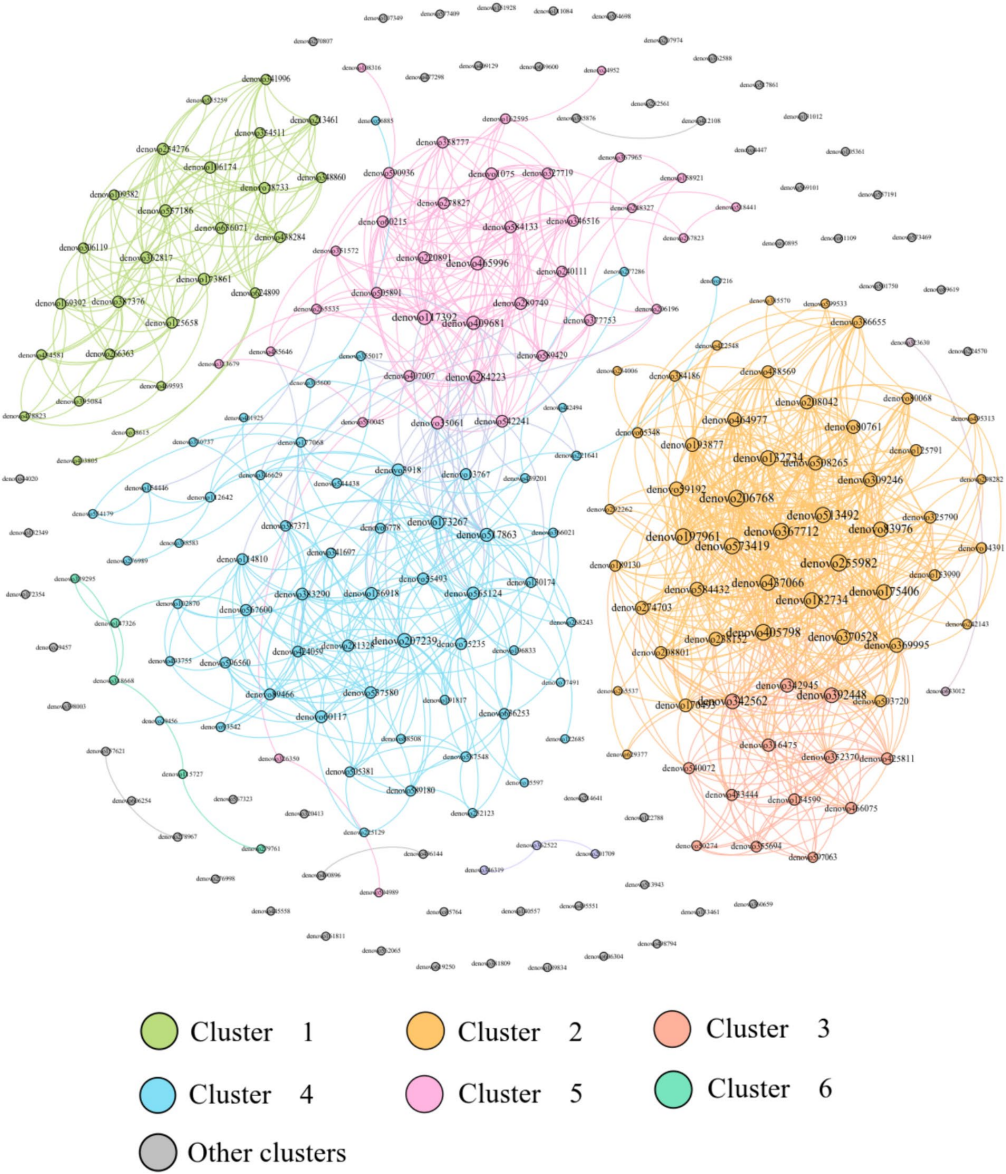
All group advantage OTU co-occurrence network.

This network analysis included the top 200 OTUs with a relative abundance and constructed a network with 200 nodes and 1,198 edges, with 1,198 positive edges and 0 negative edges, which showed that the dominant OTU co-occurrence network has a cooperative relationship dominated by microorganisms (Figure 9).

## 4 Discussion

### 4.1 Gut microorganism composition

A higher Firmicutes/Bacteroidetes (F/B) ratio has been found to facilitate the digestion of indigestible cellulose and hemicellulose in phytophagous animals such as horses and rabbits (Edgar et al., 2011). Research indicates that *Clostridiales* aids in the breakdown of cellulose and hemicellulose, while S24-7 is linked to complex carbohydrates and can enhance carbohydrate utilization in animals (Van Dyke and McCarthy, 2002; Ormerod et al., 2016). In the current study, the gut microbiota of *E. miletus* at the phylum level was predominantly composed of Firmicutes and Bacteroidetes, with the most prevalent genera being *Hologeometricum* of Euryarchaeota, S24-7 of Bacteroidetes, and Firmicutes of *Clostridiales*. This composition suggested that the gut microbiota of *E. miletus* is well suited for phytophagy, consistent with previous research (Yan et al., 2022). The F/B ratio was lower in the HF group compared to the control and Re groups, possibly due to the lower fiber content in high-fat diets, which may not necessitate a higher F/B ratio (Zhang and Yang, 2016).

### 4.2 Effect of high-fat food and different altitudes on gut microorganisms and physiological indices

High-fat diets are commonly utilized to induce obesity, and *E. miletus* subjected to a high-fat diet for 1 week exhibited a notable increase in body mass, which subsequently returned to control levels following re-feeding, indicating a high degree of adaptability. The consumption of high-fat foods led to elevated body weight and food calories as well as a decrease in RMR. Additionally, the ingestion of high-fat foods resulted in an augmentation of WAT, BAT masses, and Tg content, mirroring the changes observed in body mass. The metabolism of the high-fat diet group primarily relied on lipid-based processes, as evidenced by a significant rise in leptin levels within the high-fat diet group, suggesting heightened lipid metabolism in response to high-fat foods. While mice on high-fat diets often experience disruptions in blood glucose and lipid levels, notably, blood glucose and cholesterol levels did not exhibit significant alterations in this study, potentially due to the duration of high-fat food consumption, necessitating further investigation in subsequent studies (Neyrinck et al., 2016). It is noteworthy that the cecum of *E. miletus* from DL was shortened after the high-fat diet. Studies have shown that the cecum is a fermentation site for cellulose that can respond to changes in food quality, so the cecum of *E. miletus* from DL showed more sensitivity to high-fat food (Liu and Wang, 2007).

The stability and metabolic capacity of the host are influenced by the diversity of gut microbes, with a greater diversity indicating a heightened ability to utilize various metabolic pathways (Fändriks, 2017). The richness and diversity of the gut microflora can be assessed through the analysis of gut microbial α diversity. In the present investigation, while there was no notable distinction in the Chao index across all groups, the Shannon index in the XGRe group exhibited lower values compared to the DLHF and XGHF groups, potentially due to adaptation to a high-fat diet. Results from the PERMANOVA indicated significant disparities in the gut microbial community structure between the control and high-fat diet groups, as well as between the high-fat diet and resistant starch groups within the same region, suggesting that consumption of high-fat food can modify the gut microbial community structure in *E. miletus*. Previous research has demonstrated that high-fat diets can impact the diversity of intestinal flora, a finding that aligns with the outcomes of our study and that distinct dietary components may lead to specific microbial diversity patterns (Daniel et al., 2014).

The specificity of gut microorganisms in the host was linked to an increase in food nutrients, leading to the depletion or reorganization of bacteria (Kremer et al., 2013). For instance, *E. miletus* of the control group in DL exhibited an enrichment of microbial genera such as *Clostridium*, *Oscillospira*, and *Butyricimonas*. *Clostridium* is known to possess genes encoding fibrous and vegetative hemicellulases crucial for breaking down plant polysaccharides in ruminal animals (Dai et al., 2015). *Butyricimonas* are beneficial bacteria that produce butyric acid, along with various enzymes and nutrients that impact host metabolic conditions, inhibit harmful bacteria, and promote the growth of beneficial bacteria (Duan et al., 2019). In the XGC group, *Anaerostipes*, *Anaeroplasma*, *Desulfovibrio*, *Candidatus Arthromitus*, and *Butyricimomas* were found to be enriched. *Anaerostipes* have the ability to ferment and metabolize indigestible plant polysaccharides to provide cellular energy, while *Anaeroplasma* is primarily involved in cellulose digestion and reducing inflammation-related factors (Javier et al., 2016; Liang et al., 2018). *Desulfovibrio* can produce LPS, influencing probiotic colonization, and high levels of *Desulfovibrio* may harm intestinal epithelial cells and the intestinal barrier (Chen et al., 2018). *Candidatus Arthromitus* and *Butyricimomas* are probiotic bacteria associated with immune system functions and metabolism (Bolotin et al., 2014). In the DLHF group, *Lactobacillus* and *Hylemonella* were predominantly enriched. *Lactobacillus* has been shown to regulate the expression of receptors linked to fat absorption and plays a significant role in reducing blood lipids and glucose levels (Gan et al., 2020; Lv et al., 2021). The DLRe group exhibited enrichment in *Parabacteroides* and *Prevotella*, which support host metabolism, increase adipose tissue thermogenesis, reduce inflammation, and combat insulin resistance. *Prevotella* is positively correlated with *Clostridium* abundance and is associated with cellulose digestion and catabolism (Dai et al., 2015; Wu et al., 2019). XGRe was mainly enriched in *Anaerostipes* and *Desulfovibrio*. These findings suggested that *E. miletus* in the control group harbored flora involved in the breakdown of plant polysaccharides and cellulose, with a shift in intestinal flora toward aiding lipid metabolism to supply energy post high-fat-diet consumption. Following re-feeding, the intestinal flora transitions back to a predominantly fiber-digesting flora, with the presence of probiotics promoting lipolysis and assisting in regulating obesity induced by high-fat diets, counteracting the inflammatory response triggered by such dietary habits.

The association between physiological markers and microorganisms revealed a negative correlation between Bacteroidaceae and leptin in DL, as well as TNF-α in XGLL. Research has indicated that *Bacteroides* aids in the digestion of carbohydrates within the host and produces primarily SCFAs (Turnbaugh et al., 2006). It has been observed that a diet high in fat can decrease the presence of *Bacteroides* in the body and that obese individuals tend to harbor gut microbes that are more efficient at extracting energy from their diet compared to lean individuals (Wexler, 2007). Additionally, some studies have shown an increase in Bacteroides levels following weight loss in obese individuals (Turnbaugh et al., 2006; Wexler, 2007). Leptin plays a role in regulating the body’s energy balance by reducing food intake, increasing energy expenditure, and inhibiting fat synthesis (González Jiménez et al., 2010). It is suggested that a decrease in leptin levels may be compensated for by an increase in Bacteroidaceae during the re-feeding process after a high-fat diet in *E. miletus*, thus achieving a balance in energy capacity (González Jiménez et al., 2010). *Bacteroides fragilis* within the *Bacteroides* genus can modulate inflammatory factors, stimulate T-cell immunity, and potentially regulate the inflammatory response induced by high-fat diets in *E. miletus* (Weiss, 2002). Lachnospiraceae exhibited a positive correlation with leptin in DL and BAT mass in XGLL. Studies have shown that Lachnospiraceae ferment plant polysaccharides into SCFAs, thereby regulating energy supply and immunity and it is hypothesized that Lachnospiraceae help the *E. miletus* digest food for energy (Pascale et al., 2018). *Cholesterol* and S27-4(UG) displayed a negative correlation with Paraprevotellaceae in *E. miletus* in DL, while WAT mass was negatively associated with Paraprevotellaceae in XGLL. Studies have indicated that S27-4(UG) breaks down plant fiber and Paraprevotellaceae selectively absorbs SCFAs, providing increased energy to the host (Ormerod et al., 2016; Abbas et al., 2020). As high-fat diets lead to an increase in WAT mass and cholesterol, the abundance of S27-4(UG) and Paraprevotellaceae may decline, suggesting that *E. miletus* can regulate gut microbe populations to extract energy from various foods and maintain energy balance. *Halogeometricum* exhibited a positive correlation with leptin and cholesterol in DL, as well as leptin and triglycerides in XGLL. Although *Halogeometricum* is commonly found in mammalian gut microbes, it is typically present in low quantities. The current findings suggest that *Halogeometricum* may assist the body in extracting more energy from consumed food. Tg, Tc, leptin, and TNF-α were positively correlated with denovo624899 abundance, indicating that denovo624899 in *E. miletus* may have a preference for high-fat foods, potentially contributing to obesity under conditions of high-fat food consumption. The study results suggested that specific gut microbial flora may play a role in regulating energy metabolism and immunity in response to high-fat diets, thereby influencing the adaptation of *E. miletus* to varying altitudes.

### 4.3 Regional differences in body mass regulation under the influence of high-fat food

The study observed no increase in food intake in DL and XGLL of *E. miletus* when acclimated to high-fat diets, but the increase in food calories in the high-fat groups suggested that the increase of body mass may be related to the increase in energy intake. The species appeared to adjust its metabolism in response to improved food and environmental conditions. Higher levels of leptin, a hormone that suppresses appetite, and a decrease in Bacteroidetes, a type of gut bacteria associated with reduced food intake, were noted (González Jiménez et al., 2010; Mack et al., 2016). Leptin levels were higher in DL compared to XGLL, correlating with the significantly greater body mass in DL. Additionally, leptin levels were significantly elevated in the high-fat group compared to other groups, while this group exhibited a lower proportion of Bacteroidetes, potentially explaining the lack of increased food intake. These findings suggest regional variations in the physiological regulation of *E. miletus* between the two regions.

Previous research has demonstrated that variations in gut microbiota in *E. miletus* can be linked to dietary resources. This current experimental investigation revealed that the impact of a high-fat diet on the gut microbiota of *E. miletus* from different regions varied. The Venn diagram results indicated that in the control group, DL exhibited fewer intestinal microbial genera compared to XGLL. However, after being subjected to a high-fat diet, DL showed an increase in microbial genera while XGLL exhibited a decrease. Following a period of re-feeding, DL once again displayed fewer microbial genera than XGLL. The interplay between environmental factors and host gut microbiota is closely associated with dietary patterns. It is postulated that varying food availability and composition influence the energy intake of *E. miletus*, consequently impacting the composition of gut flora. As food diversity increases, the diversity of bacteria and functional genera in the host gut microbiota also increases (Yan and Zhu, 2023). Compared with the *E. miletus* in DL, XGLL has a higher altitude and a lower temperature in winter. Studies have shown that food diversity was higher in XGLL than in DL and that gut microbial diversity correlates with food diversity so that XGLL has more genera of gut microbes in order to assist *E. miletus* in obtaining more energy and improve its adaptation in winter (Yan and Zhu, 2023). Analysis of co-occurrence networks suggested that gut microbes in *E. miletus* exhibit cooperative relationships, with positive cooperativity facilitating the adaptation of *E. miletus* to diverse environmental challenges (Li et al., 2019).

## 5 Conclusion

The current research was the first to examine the effects of a high-fat diet on the intestinal microbiota of *E. miletus* across different altitudes. The findings revealed that high-fat foods led to alterations in gut microbiota diversity and specific microbial abundance in *E. miletus*, with both commonalities and differences observed in the changes of gut microbial organisms between the two regions. Parameters related to energy regulation such as body mass, BAT mass, WAT mass, leptin, and Tg were influenced by a high-fat diet and showed correlations with the abundance of *Halogeometricum*, *Lachnospiraceae (UG)*, *Paraprevotellaceae (UG)*, and *Bacteroidaceae (UG)* in *E. miletus*. The interaction between gut microbes and physiological indices of *E. miletus* on a high-fat diet played a role in digestion and the regulation of energy metabolism and immunity through signaling molecules and metabolites. The adaptability of the gut microbiota composition in *E. miletus*, along with a diverse metabolic pool of microorganisms, facilitated a swift transition to a new dietary ecological niche.

## Data availability statement

The datasets presented in this study can be found in online repositories. The names of the repository/repositories and accession number(s) can be found at: https://www.ebi.ac.uk/ena, PRJEB61600 and https://doi.org/10.6084/m9.figshare.24313918.v1, 24313918.v1.

## Ethics statement

All animal operation procedures comply with the rules of Animals Care and Use Committee of School of Life Sciences, Yunnan Normal University. This study was approved by the Committee (13-0901-011). The study was conducted in accordance with the local legislation and institutional requirements.

## Author contributions

TJ: Investigation, Methodology, Writing – original draft. WeZ: Investigation, Methodology, Software, Writing – original draft. LC: Methodology, Investigation, Writing – original draft. WaZ: Funding acquisition, Writing – original draft, Writing – review & editing. LF: Funding acquisition, Writing – review & editing.

## Footnotes

^1^ https://bioinfogp.cnb.csic.es/tools/venny/index.html

## References

[ref1] Abbas W. Howard J. T. Paz H. A. Hales K. E. Wells J. E. Kuehn L. A.. (2020). Influence of host genetics in shaping the rumen bacterial community in beef cattle. Sci. Rep. 10:15101. doi: 10.1038/s41598-020-72011-9, PMID: 32934296 PMC7493918

[ref2] Amato K. R. Yeoman C. J. Kent A. Righini N. Carbonero F. Estrada A.. (2013). Habitat degradation impacts black howler monkey (*Alouatta pigra*) gastrointestinal microbiomes. ISME J. 7, 1344–1353. doi: 10.1038/ismej.2013.16, PMID: 23486247 PMC3695285

[ref3] Bolnick D. I. Snowberg L. K. Hirsch P. E. Lauber C. L. Knight R. Caporaso J. G.. (2014). Individuals' diet diversity influences gut microbial diversity in two freshwater fish (three spine stickleback and Eurasian perch). Ecol. Lett. 17, 979–987. doi: 10.1111/ele.12301, PMID: 24847735 PMC4084827

[ref4] Bolotin A. de Wouters T. Schnupf P. Bouchier C. Loux V. Rhimi M.. (2014). Genome sequence of “*Candidatus arthromitus*” sp. strain SFB-mouse-NL, a commensal bacterium with a key role in postnatal maturation of gut immune functions. Genome Announc. 2, e00705–e00714. doi: 10.1128/genomeA.00705-14, PMID: 25035333 PMC4102870

[ref5] Carmody R. N. Gerber G. K. Luevano J. M. Gatti D. M. Somes L. Svenson K. L.. (2015). Diet dominates host genotype in shaping the murine gut microbiota. Cell Host Microbe 17, 72–84. doi: 10.1016/j.chom.2014.11.010, PMID: 25532804 PMC4297240

[ref6] Chen Y. F. Jin L. Li Y. H. Xia G. B. Chen C. Zhang Y. (2018). Bamboo-shaving polysaccharide protects against high-diet induced obesity and modulates the gut microbiota of mice. J. Funct. Foods 49, 20–31. doi: 10.1016/j.jff.2018.08.015

[ref7] Dai X. Tian Y. Li J. T. Luo Y. F. Liu D. Zheng H. J.. (2015). Metatranscriptomic analyses of plant cell wall polysaccharide degradation by microorganisms in the cow rumen. Appl. Environ. Microbiol. 81, 1375–1386. doi: 10.1128/AEM.03682-14, PMID: 25501482 PMC4309707

[ref8] Daniel H. Gholami A. M. Berry D. Desmarchelier C. Hahne H. Loh G.. (2014). High-fat diet alters gut microbiota physiology in mice. ISME J. 8, 295–308. doi: 10.1038/ismej.2013.155, PMID: 24030595 PMC3906816

[ref9] David L. A. Maurice C. F. Carmody R. N. Gootenberg D. B. Button J. E. Wolfe B. E.. (2014). Diet rapidly and reproducibly alters the human gut microbiome. Nature 505, 559–563. doi: 10.1038/nature12820 24336217 PMC3957428

[ref10] Duan Y. F. Zhang J. S. Huang J. H. Jiang S. G. (2019). Effects of dietary *clostridium butyricum* on the growth, digestive enzyme activity, antioxidant capacity, and resistance to nitrite stress of *Penaeus monodon*. Probiotics Antimicrob Proteins 11, 938–945. doi: 10.1007/s12602-018-9421-z, PMID: 29858778

[ref11] Edgar R. C. Haas B. J. Clemente J. C. Quince C. Knight R. (2011). UCHIME improves sensitivity and speed of chimera detection. Bioinformatics 27, 2194–2200. doi: 10.1093/bioinformatics/btr381, PMID: 21700674 PMC3150044

[ref12] Fändriks L. (2017). Roles of the gut in the metabolic syndrome: an overview. J. Intern. Med. 281, 319–336. doi: 10.1111/joim.12584 27991713

[ref13] Gan Y. Tang M. W. Tan F. Zhou X. R. Fan L. Xie Y. X.. (2020). Anti-obesity effect of *Lactobacillus plantarum* CQPC01 by modulating lipid metabolism in high-fat diet-induced C57BL/6 mice. J. Food Biochem. 44:e13491. doi: 10.1111/jfbc.13491 33006202

[ref14] Gong X. N. Jia T. Zhang D. Wang Z. K. (2021). Faster response to high-fat diet in body mass regulation from lower altitude population in *Eothenomys miletus* from Hengduan mountain regions. Pakistan. J. Zool. 54:216. doi: 10.17582/journal.pjz/20200211050216

[ref15] Gong Z. D. Wu H. Y. Duan X. D. Feng X. G. Zhang Y. Z. Liu Q. (2001). The species diversity and distribution trends of small mammals in Hengduan Mountains, Yunnan. Biodivers. Sci. 9, 73–79. doi: 10.3321/j.issn:1005-0094.2001.01.011

[ref16] González Jiménez E. Aguilar Cordero M. J. García García C. d. J. García López P. A. Álvarez Ferre J. Padilla López C. A. (2010). Leptina: un péptido con potencial terapéutico en sujetos obesos [leptin: a peptide with therapeutic potential in the obese]. Endocrinol. Nutr. 57, 322–327. doi: 10.1016/j.endonu.2010.03.018, PMID: 20605117

[ref17] Guo N. Wu Q. F. Shi F. Y. Niu J. H. Zhang T. Degen A. A.. (2021). Seasonal dynamics of diet-gut microbiota interaction in adaptation of yaks to life at high altitude. NPJ Biofilms Microbiomes 7:38. doi: 10.1038/s41522-021-00207-6, PMID: 33879801 PMC8058075

[ref18] Javier F. Saúl R. B. Ignacio G. R. Elisa M. M. Claudoi J. V. Felipe L. (2016). Colon microbiota fermentation of dietary prebiotics towards short-chain fatty acids and their roles as anti-inflammatory and antitumour agents: a review. J. Funct. Foods 25, 511–522. doi: 10.1016/j.jff.2016.06.032

[ref19] Kong C. Gao R. Y. Yan X. B. Huang L. S. Qin H. L. (2019). Probiotics improve gut microbiota dysbiosis in obese mice fed a high-fat or high-sucrose diet. Nutrition 60, 175–184. doi: 10.1016/j.nut.2018.10.002 30611080

[ref20] Konrad D. Wueest S. (2014). The gut-adipose-liver axis in the metabolic syndrome. Physiology 29, 304–313. doi: 10.1152/physiol.00014.2014, PMID: 25180260

[ref21] Kremer N. Philipp E. E. Carpentier M. C. Brennan C. A. Kraemer L. Altura M. A.. (2013). Initial symbiont contact orchestrates host-organ-wide transcriptional changes that prime tissue colonization. Cell Host Microbe 14, 183–194. doi: 10.1016/j.chom.2013.07.006, PMID: 23954157 PMC3928804

[ref22] Li G. L. Li J. Kohl K. D. Yin B. F. Wei W. H. Wan X. R.. (2019). Dietary shifts influenced by livestock grazing shape the gut microbiota composition and co-occurrence networks in a local rodent species. J. Anim. Ecol. 88, 302–314. doi: 10.1111/1365-2656.12920, PMID: 30381827

[ref23] Liang Y. J. Zhang Y. P. Deng Y. J. Liang S. He Y. F. Chen Y. N.. (2018). Chaihu-Shugan-san decoction modulates intestinal microbe dysbiosis and alleviates chronic metabolic inflammation in NAFLD rats via the NLRP3 inflammasome pathway. Evid. Based Complement. Alternat. Med. 2018:9390786. doi: 10.1155/2018/9390786, PMID: 30105078 PMC6076928

[ref24] Liu Q. S. Wang D. H. (2007). Effects of diet quality on phenotypic flexibility of organ size and digestive function in Mongolian gerbils (*Meriones unguiculatus*). J Compar Physiol B Biochem Syst Environ Physiol 177, 509–518. doi: 10.1007/s00360-007-0149-4, PMID: 17333208

[ref25] Lucchini F. C. Wueest S. Challa T. D. Item F. Modica S. Borsigova M.. (2020). ASK1 inhibits browning of white adipose tissue in obesity. Nat. Commun. 11:1642. doi: 10.1038/s41467-020-15483-7, PMID: 32242025 PMC7118089

[ref26] Lv X. C. Chen M. Huang Z. R. Guo W. L. Ai L. Z. Bai W. D.. (2021). Potential mechanisms underlying the ameliorative effect of *Lactobacillus paracasei* FZU103 on the lipid metabolism in hyperlipidemic mice fed a high-fat diet. Food Res. Int. 139:109956. doi: 10.1016/j.foodres.2020.109956, PMID: 33509508

[ref27] Mack I. Cuntz U. Grämer C. Niedermaier S. Pohl C. Schwiertz A.. (2016). Weight gain in anorexia nervosa does not ameliorate the faecal microbiota, branched chain fatty acid profiles, and gastrointestinal complaints. Sci. Rep. 6:26752. doi: 10.1038/srep26752, PMID: 27229737 PMC4882621

[ref28] Maurice C. F. Knowles S. C. Ladau J. Pollard K. S. Fenton A. Pedersen A. B.. (2015). Marked seasonal variation in the wild mouse gut microbiota. ISME J. 9, 2423–2434. doi: 10.1038/ismej.2015.53, PMID: 26023870 PMC4611506

[ref29] Moran N. A. Ochman H. Hammer T. J. (2019). Evolutionary and ecological consequences of gut microbial communities. Annu. Rev. Ecol. Evol. Syst. 50, 451–475. doi: 10.1146/annurev-ecolsys-110617-062453, PMID: 32733173 PMC7392196

[ref30] Neyrinck A. M. Schüppel V. L. Lockett T. Haller D. Delzenne N. M. (2016). Microbiome and metabolic disorders related to obesity: which lessons to learn from experimental models? Trends Food Sci. Technol. 57, 256–264. doi: 10.1016/j.tifs.2016.08.012

[ref31] Ormerod K. L. Wood D. L. Lachner N. Gellatly S. L. Daly J. N. Parsons J. D.. (2016). Genomic characterization of the uncultured Bacteroidales family S24-7 inhabiting the guts of homeothermic animals. Microbiome 4:36. doi: 10.1186/s40168-016-0181-2, PMID: 27388460 PMC4936053

[ref32] Pascale A. Marchesi N. Marelli C. Coppola A. Luzi L. Govoni S.. (2018). Microbiota and metabolic diseases. Endocrine 61, 357–371. doi: 10.1007/s12020-018-1605-5 29721802

[ref33] Percie du Sert N. Ahluwalia A. Alam S. Avey M. T. Baker M. Browne W. J.. (2020). Reporting animal research: explanation and elaboration for the ARRIVE guidelines 2.0. PLoS Biol. 18:e3000411. doi: 10.1371/journal.pbio.3000411, PMID: 32663221 PMC7360025

[ref34] Sender R. Fuchs S. Milo R. (2016). Revised estimates for the number of human and bacteria cells in the body. PLoS Biol. 14:e1002533. doi: 10.1371/journal.pbio.1002533, PMID: 27541692 PMC4991899

[ref35] Steelman S. M. Chowdhary B. P. Dowd S. Suchodolski J. Janečka J. E. (2012). Pyrosequencing of 16S rRNA genes in fecal samples reveals high diversity of hindgut microflora in horses and potential links to chronic laminitis. BMC Vet. Res. 8:231. doi: 10.1186/1746-6148-8-231, PMID: 23186268 PMC3538718

[ref36] Tan B. L. Norhaizan M. E. (2019). Effect of high-fat diets on oxidative stress, cellular inflammatory response and cognitive function. Nutrients 11:2579. doi: 10.3390/nu11112579, PMID: 31731503 PMC6893649

[ref37] Thaiss C. A. Zmora N. Levy M. Elinav E. (2016). The microbiome and innate immunity. Nature 535, 65–74. doi: 10.1038/nature18847 27383981

[ref38] Tremaroli V. Kovatcheva-Datchary P. Bäckhed F. (2010). A role for the gut microbiota in energy harvesting? Gut 59, 1589–1590. doi: 10.1136/gut.2010.223594 20940281

[ref39] Turnbaugh P. J. Ley R. E. Mahowald M. A. Magrini V. Mardis E. R. Gordon J. I. (2006). An obesity-associated gut microbiome with increased capacity for energy harvest. Nature 444, 1027–1031. doi: 10.1038/nature05414, PMID: 17183312

[ref40] Van Dyke M. I. McCarthy A. J. (2002). Molecular biological detection and characterization of Clostridium populations in municipal landfill sites. Appl. Environ. Microbiol. 68, 2049–2053. doi: 10.1128/AEM.68.4.2049-2053.2002, PMID: 11916731 PMC123838

[ref41] Weiss S. T. (2002). Eat dirt--the hygiene hypothesis and allergic diseases. N. Engl. J. Med. 347, 930–931. doi: 10.1056/NEJMe020092 12239263

[ref42] Wexler H. M. (2007). Bacteroides: the good, the bad, and the nitty-gritty. Clin. Microbiol. Rev. 20, 593–621. doi: 10.1128/CMR.00008-07, PMID: 17934076 PMC2176045

[ref43] Wu T. R. Lin C. S. Chang C. J. Lin T. L. Martel J. Ko Y. F.. (2019). Gut commensal *Parabacteroides goldsteinii* plays a predominant role in the anti-obesity effects of polysaccharides isolated from *Hirsutella sinensis*. Gut 68, 248–262. doi: 10.1136/gutjnl-2017-315458, PMID: 30007918

[ref44] Yan B. W. Jia T. Wang Z. K. Zhu W. L. (2022). Comparative research of intestinal microbiota diversity and body mass regulation in *Eothenomys miletus* from different areas of Hengduan mountain regions. Front. Microbiol. 13:1026841. doi: 10.3389/fmicb.2022.1026841, PMID: 36325022 PMC9619095

[ref45] Yan B. W. Zhu W. L. (2023). Research on feeding habits and stomach fungi in *Eothenomys miletus* from Hengduan mountain regions. Life Res 6:11. doi: 10.53388/LR20230011

[ref46] Yin J. Li Y. Y. Han H. Chen S. Gao J. Liu G.. (2018). Melatonin reprogramming of gut microbiota improves lipid dysmetabolism in high-fat diet-fed mice. J. Pineal Res. 65:e12524. doi: 10.1111/jpi.12524, PMID: 30230594

[ref47] Zhang M. Yang X. J. (2016). Effects of a high fat diet on intestinal microbiota and gastrointestinal diseases. World J. Gastroenterol. 22, 8905–8909. doi: 10.3748/wjg.v22.i40.8905, PMID: 27833381 PMC5083795

[ref48] Zhou D. Pan Q. Xin F. Z. Zhang R. N. He C. X. Chen G. Y.. (2017). Sodium butyrate attenuates high-fat diet-induced steatohepatitis in mice by improving gut microbiota and gastrointestinal barrier. World J. Gastroenterol. 23, 60–75. doi: 10.3748/wjg.v23.i1.60, PMID: 28104981 PMC5221287

[ref49] Zhu W. L. Cai J. H. Lian X. Wang Z. K. (2011). Effects of photoperiod on energy intake, thermogenesis and body mass in *Eothenomys miletus* in Hengduan Mountain region. J. Therm. Biol. 36, 380–385. doi: 10.1016/j.jtherbio.2011.06.014

[ref50] Zhu W. L. Jia T. Lian X. Wang Z. K. (2010). Effects of cold acclimation on body mass, serum leptin level, energy metabolism and thermognesis in *Eothenomys miletus* in Hengduan Mountains region. J. Therm. Biol. 35, 41–46. doi: 10.1016/j.jtherbio.2009.10.006

[ref51] Zhu W. L. Mu Y. Zhang H. Gao W. R. Zhang L. Wang Z. K. (2014b). Effects of random food deprivation on body mass, behavior and serum leptin levels in *Eothenomys miletus* (Mammalia: Rodentia: Cricetidae). Ital J Zool 81, 227–234. doi: 10.1080/11250003.2014.902511

[ref52] Zhu L. Wu Q. Dai J. Zhang S. Wei F. (2011). Evidence of cellulose metabolism by the giant panda gut microbiome. Proc. Natl. Acad. Sci. U. S. A. 108, 17714–17719. doi: 10.1073/pnas.1017956108, PMID: 22006317 PMC3203778

[ref53] Zhu W. L. Yang Y. H. Jia T. Lian X. Wang Z. K. Gong Z. D.. (2008). Evaporative water loss and body temperature regulation in *Eothenomys miletus* and *Apodemus chevrieri*. Acta Theriol Sin. 28, 65–74. doi: 10.16829/j.slxb.2008.01.011

[ref54] Zhu W. L. Yang S. C. Zhang L. Wang Z. K. (2012). Seasonal variations of body mass, thermogenesis and digestive tract morphology in *Apodemus chevrieri* in Hengduan mountain region. Anim. Biol. 62, 463–478. doi: 10.1163/157075612X650140

[ref55] Zhu W. L. Zhang H. Zhang L. Yu T. T. Wang Z. K. (2014a). Thermogenic properties of Yunnan red-backed voles (*Eothenomys miletus*) from the Hengduan mountain region. Anim. Biol. 64, 59–73. doi: 10.1163/15707563-00002430

